# Urinary complement profile in IgA nephropathy and its correlation with the clinical and pathological characteristics

**DOI:** 10.3389/fimmu.2023.1117995

**Published:** 2023-03-20

**Authors:** Dongqing Wang, Changwei Wu, Sipei Chen, Yi Li, Li Wang, Yong Zhang, Guisen Li

**Affiliations:** ^1^ Renal Department and Nephrology Institute, Sichuan Provincial People’s Hospital, School of Medicine, University of Electronic Science and Technology of China, Chengdu, China; ^2^ Institutes for Systems Genetics, West China Hospital, Sichuan University, Chengdu, China

**Keywords:** IgA nephropathy, complement, proteomics, histopathology, proteinuria

## Abstract

**Background and objectives:**

The activated complement profile in IgA nephropathy (IgAN) is still unclear. Our study investigated the profile of urinary complements in IgAN patients and its correlations with clinical and pathological characteristics.

**Methods:**

Urinary protein abundance was detected by liquid chromatography-tandem mass spectrometry (LC–MS/MS) in 50 IgAN, 50 membranous nephropathy (MN), and 68 healthy controls (HC). Then, Gene Ontology (GO) and Kyoto Encyclopedia of Genes and Genomes (KEGG) analyses were performed to identify differentially expressed proteins in IgAN patients. The differentially expressed complement proteins were screened in IgAN patients, and their correlations with laboratory or pathological parameters were analyzed. Thereafter, 7 complement components were validated by enzyme-linked immunosorbent assay (ELISA) in the urine samples of 45 IgAN patients.

**Results:**

There were 786 differentially expressed proteins between IgAN and HC. KEGG analysis showed that differentially expressed urinary proteins in IgAN were enriched with complement. Of these, 67% of urinary complement protein abundance was associated with the estimated glomerular filtration rate. The urinary complement-related protein collectin12 (colec12), complement H factor (CFH), complement H factor-related protein 2 (CFHR2), and complement B factor (CFB) were positively correlated with serum creatinine; colec12, CFHR2, CFB, and C8g were positively correlated with glomerulosclerosis; CFH, CFHR2, C8g, and C9 were positively correlated with tubular atrophy/interstitial fibrosis.

**Conclusion:**

Abnormally increased components of complement pathways significantly correlate with reduced renal function, proteinuria, and renal histological damage in IgAN. It could provide a potential biomarker panel for monitoring IgAN and provide clues for therapeutic choice targeting complement system of IgAN patients.

## Introduction

IgA nephropathy (IgAN) is one of the most common glomerular diseases, and activation of the complement pathways plays an important role in its pathogenesis (1–6). Immune complexes containing IgA1 and autoantibodies are deposited in the kidney, activating complements and causing immune inflammation, finally leading to mesangial cell and tissue injury (1–3). Several early studies have confirmed that multiple components of complement system are positive in biopsy kidney tissues of IgAN, including C3, properdin, C4b Binding Protein (C4BP), etc. (3, 4). Renal biopsies of patients with IgAN showed glomerular deposition of mannose-binding lectin (MBL), L-ficolin, and MBL-associated serine proteases (MASP) (7). Collectin 11, an initiator of the complement lectin pathway, was involved in both acute kidney injury and chronic tubulointerstitial fibrosis (8). It suggested the involvement of the alternative and lectin pathways in the pathogenesis of IgAN (2–6, 9).

Increasing evidence has shown that the activated serum or glomerular complements were related to the clinical characteristics of IgAN patients (proteinuria, hematuria, renal function) and renal pathological changes (2–6). Urinary C3a and C5a levels correlated with crescents (10). Furthermore, glomerular deposition of FHR5, C3, C3dg, C4d, C5b9, and collectin 11 was associated with IgAN progression (8, 11–13). The arteriolar C4d in IgAN kidney biopsy tissue was associated with mean arterial pressure, chronic microangiopathy, and disease progression (14). Complement factor H (CFH) and complement factor H-related protein genes (CFHRs) were also investigated. CFHR-5 was associated with kidney histological severity (15, 16) and serum CFHR-5 was independently associated with the progression of IgAN (12, 15).

Previous studies have often focused on one or several complement components. The different positive rates of different complement proteins in serum, glomeruli, or mesangial areas of IgAN patients might be influenced by the disease status or treatment (13). There is a lack of data about complement activation profile of IgAN patients. The progression in liquid chromatography-tandem mass spectrometry (LC–MS/MS)-based proteomic studies provides an important opportunity for systematic complement analysis. Our previous studies demonstrated that it could reveal the characteristics of proteome and protein glycosylation (17, 18), and provide noninvasive measures for differential diagnosis of kidney diseases (19). The high-quality proteomic assay based on mass spectrometry can reveal a complete profile of the complement activation in IgAN patients.

Therefore, we used proteomic analysis to detect the complements in the urine and analyze their correlation with the clinical and pathological manifestations of IgAN patients. In this study, we first performed liquid chromatography-tandem mass spectrometry analysis of urine proteins from patients with different renal diseases as well as healthy controls, to obtain urine complement profile in patients with IgAN. The correlation between complement activation and clinical or pathological parameters in IgAN patients was analyzed. Then, an enzyme-linked immunosorbent assay (ELISA) was used to validate the complement components in an isolated IgAN group. It is expected to provide information for the monitoring and treatment of IgAN patients, especially for complement-targeted therapy.

## Materials and methods

This is a concise version. Please check the **Supplementary Materials** for the complete version.

### Participants and study design

In this study, a total of 213 participants were recruited from 2019 to 2022 at Sichuan Provincial People’s Hospital. A total of 213 urine samples from subjects with IgA nephropathy (IgAN) (n = 95), membranous nephropathy (MN, n = 50), and healthy controls (HC) (n = 68) were collected in tubes according to standard operating procedures. Urine samples of patients were collected on the morning of renal biopsy. All IgAN patients were diagnosed as a primary entity, excluding secondary IgAN, such as secondary to systemic lupus erythematosus, cirrhosis, rheumatoid arthritis, and Henoch-Schönlein purpura nephritis. The subjects were divided into the discovery cohort, including 50 IgAN patients, 50 MN patients, and 68 HC for urine proteomics analysis, and the validation cohort, including an isolated group of 45 IgAN for ELISA urine testing. The study complied with the Declaration of Helsinki principles and was approved by the medical ethics committees of Sichuan Provincial People’s Hospital. Written informed consent was obtained from all participants.

Clinical, laboratory, and pathological parameters were collected, and estimated glomerular filtration rate (eGFR) was calculated by using the CKD-EPI formula (20). We evaluated pathological features based on the revised Oxford system of classification of IgAN (21).

### Urinary protein digestion and mass spectrometric analysis

The clean midstream urine samples were collected in the morning for renal biopsy and centrifuged for 20 min at 1000× g. The 500 μL supernatant was collected in 1.5 ml tubes and then stored at −80 °C. At room temperature, frozen urine samples were thawed and gently homogenized before analysis. The urinary protein digestion, mass spectrometric analysis, and spectral establishment used in this study were described in our previous report (19). Urinary peptide analysis was analyzed by liquid chromatography-tandem mass spectrometry and performed using an Orbitrap Fusion Lumos mass spectrometer (Thermo Fisher Scientific, Waltham, MA, USA).

The raw mass spectrometric data were converted into mascot generic format (MGF) files. The x-coordinate was the mass-to-charge ratio (m/z), and the y-coordinate was the relative peak intensity. The mass spectra from each file were used to profile the urinary proteome of each patient. The urine proteins were expressed by normalization of the abundance of one protein/all proteins in one patient.

### Differentially expressed proteins, GO, and KEGG analysis

To screen the differentially expressed proteins, we used log2 (fold change) absolute values greater than 1.5, and *P* value < 0.05 (IgAN *vs.* HC, IgAN *vs.* MN, respectively). For the Gene Ontology (GO) and Kyoto Encyclopedia of Genes and Genomes (KEGG) analyses, all differentially expressed proteins were analyzed using the DAVID database (https://david.ncifcrf.gov/).

### Validation by enzyme-linked immunosorbent assay

Seven differential complement components, including C4a, C6, decay-accelerating factor (CD55), complement factor B (CFB), CFH, complement factor H related 2 (CFHR2), and collectin12 (colec12) were further detected in urine samples by the ELISA kits produced by Shanghai Zhuocai Company. All assays were performed following the manufacturer’s instructions. The frozen sample was thawed rapidly at 37°C and immediately moved to ice to prevent complement activation before dilution. After dilution, the sample was loaded into the microanalysis wells as quickly as possible. The urine sample had only one freezing/thawing cycle before analysis. The concentration of urinary complement analyzed by ELISA was normalized to the urinary protein-to-creatinine ratio.

### Statistical analysis

The correlation between the urinary complement levels and other variables was assessed using Spearman’s or Pearson’s correlation analysis. Statistics were considered significant at a two-tailed *P* value (*P* < 0.05). The statistical analyses were conducted using SPSS Statistics version 22.0 (SPSS Inc., Chicago, IL, USA).

## Results

### Baseline demographic, clinical and pathological characteristics of the patients with IgAN

There were 50 patients with IgAN in the discovery cohort and 45 patients with IgAN in the validation cohort (**Table 1**). There were no significant differences in gender, age, hypertension, serum creatinine, and eGFR between the two cohorts at the time of renal biopsy. The proteinuria (2.11 g/24 h *vs.* 1.44 g/24 h, *P* < 0.01) and the proportion of mesangial hypercellularity (72% *vs.* 29%, *P* < 0.01) in the discovery cohort were significantly higher than those in the validation cohort.

**Table 1:** Baseline demographic, clinical, and pathological characteristics of patients with IgAN.

Characteristics	Discovery cohort (n=50)	Validation cohort (n=45)	*P Va*lue
Gender (male, %)	25 (50%)^c^	27 (60%)^c^	0.328
Age (yr)	37.02 ± 14.09^a^	38.93 ± 13.74^a^	0.505
Hypertension (mmHg)	15 (30%)^c^	10 (22.2%)^c^	0.39
Serum creatinine (μmol/L)	89.05 (62.98, 127.58)^b^	106.21 ± 67.30^a^	0.571
eGFR (mL/min/1.73m^2^)	84.32 ± 36.25^a^	81.30 ± 31.89^a^	0.668
Proteinuria (g/24h)	2.11 (1.20, 3.69)^b^	1.44 ± 0.75^a^	<0.01
Hematuria	19.50 (4.26, 76.98)^b^	14.44 (4.49, 41.47)^b^	0.412
Crescents			0.123
Percentage of all patients	27 (54%)^c^	19 (42%)^c^	
Per patient (%)	5.45 (0, 20.55)^b^	0 (0, 11)^b^	
Cellular/fibrocellular crescents			0.542
Percentage of all patients	21 (42%)^c^	6 (13%)^c^	
Per patient (%)	0 (0, 10.83)^b^	0 (0, 10.5)^b^	
Fibrous crescents			0.058
Percentage of all patients	15 (30%)^c^	17 (38%)^c^	
Per patient (%)	0 (0, 5.78)^b^	0 (0, 0)^b^	
Mesangial hypercellularity n (%)	36 (72%)^c^	13 (29%)^c^	<0.01
Glomerulosclerosis			0.232
Percentage of all patients	21 (42%)^c^	25 (56%)^c^	
Per patient (%)	0 (0, 12.95)^b^	5 (0, 15)^b^	
Monocyte infiltration			0.064
Percentage of all patients	42 (84%)^c^	41 (91%)^c^	
Per patient (%)	5 (5, 5)^b^	5 (5, 6)^b^	
Tubular atrophy/interstitial fibrosis			0.707
Percentage of all patients	42 (84%)^c^	42 (93%)^c^	
Per patient (%)	5 (5, 11.25)^b^	5 (5, 10)^b^	

IgAN, immunoglobulin A nephropathy; eGFR, estimated glomerular filtration rate; Hematuria, expressed by RBC counts per high-power field. a: Data are expressed as means ± standard deviations. b: Data are expressed as medians and interquartile ranges (IQRs). c: Data are expressed as frequency and ratio.

### Urinary proteome analysis

**Figure 1:**
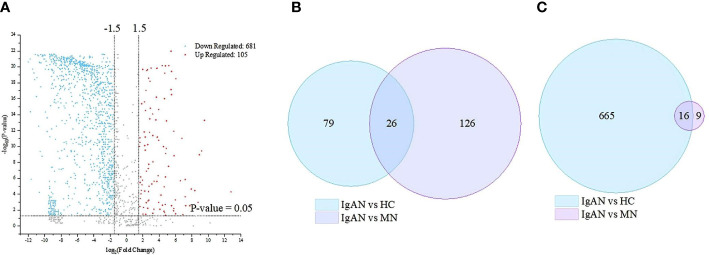
Based on mass spectrometry analysis of the discovery cohort, there are 786 and 177 differentially expressed proteins between IgA nephropathy (IgAN) and healthy controls (HC) and between IgAN and membranous nephropathy (MN), respectively. **(A)** Volcano plot of the differentially expressed proteins in the IgAN and HC. **(B)** Venn diagram of the up-regulated differential proteins in the IgAN vs. HC, and IgAN vs MN respectively. **(C)** Venn diagram of the down-regulated differential proteins in the IgAN vs HC, and IgAN vs MN respectively.

A total of 1750 proteins were identified by LC–MS/MS, 1001 in IgAN, 998 in MN, and 1686 in HC. The abundance of 786 proteins in IgAN patients was significantly different from those in HC (105 upregulated and 681 downregulated proteins). In addition, between the IgAN and MN groups, 177 proteins showed significant differences (152 upregulated and 25 downregulated proteins) (**Figure 1**).

### GO annotation and KEGG analysis of differentially expressed proteins

To further study the function of proteinuria in patients with IgAN, GO analysis was performed on 786 proteins that were significantly differentially expressed between the IgAN and HC. In the biological process (BP) category, the differentially expressed proteins were mainly related to complement activation and cell adhesion. In the cellular component (CC) category, the differentially expressed proteins were mainly associated with blood microparticles and extracellular exosomes. In the molecular function (MF) category, the differentially expressed proteins were mainly related to serine-type endopeptidase inhibitor activity and calcium ion binding. In addition, the bioinformatics analysis between IgAN and MN showed that the differentially expressed proteins were also significantly enriched in complement activation pathway.

**Figure 2:**
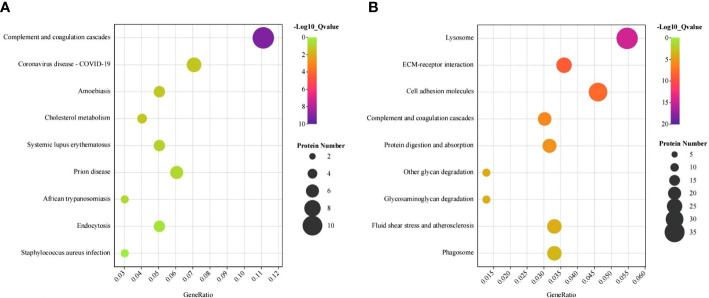
KEGG bubble plots of urinary differentially expressed proteins between IgA nephropathy (IgAN) and healthy controls (HC). KEGG bubble plots of urinary differentially expressed proteins up-regulated **(A)** and down-regulated **(B)** between IgAN and HC, respectively.

KEGG enrichment analysis showed that the differentially expressed proteins were significantly enriched in the complement, coagulation cascade pathways, and lysosomal pathways. Complement activation is one of the most abundant pathways (**Figure 2**). This pathway contained a total of 33 (4.2%) significantly differentially expressed proteins.

### Correlation between urinary complement proteins and laboratory parameters in patients with IgAN

Therefore, we focused on complement-related proteins. Of all the quantified proteins, 44 complement-related proteins were detected. The difference analysis of urinary complement proteins between IgAN, MN, and HC is shown in **Table 2**. The urinary abundance of 35 complement-related proteins was significantly different between IgAN patients and HC (*P* < 0.05). Moreover, the urinary abundance of 14 complement-associated proteins was significantly different between IgAN and MN (*P* < 0.05).

**Table 2:** Comparisons of urinary complement proteins between IgAN patients and HC or MN patients, respectively.

Urine complements	*P* values (IgAN vs HC)	*P* values IgAN vs MN	Urine complements	*P* values (IgAN vs HC)	*P* values IgAN vs MN
COLEC12	1.2×10^-14^	0.002	CPN1	5.1×10^-6^	0.785
C1R	0.001	0.992	CPN2	2.2×10^-14^	2.8×10^-4^
C1S	5.8×10^-14^	0.564	CLU	3.5×10^-16^	0.001
C2	0.85	0.066	VTN	2.9×10^-11^	0.123
C4A	0.19	0.053	CFHR1	5.4×10^-15^	0.529
C4B	0.73	6.5×10^-5^	CFHR2	0.001	0.326
MASP2	1.6×10^-18^	0.148	CFHR3	0.86	0.322
CFD	8.7×10^-5^	0.661	C3AR1	0.023	0.322
CFB	0.71	0.039	SERPINF1	0.65	0.053
CFI	7.1×10^-17^	0.004	SERPINC1	3.3×10^-14^	0.85
CFH	0.086	0.058	SERPINA1	7.2×10^-21^	1.8×10^-5^
C3	6.3×10^-12^	0.041	SERPINA3	4.1×10^-16^	0.944
C5	0.69	0.827	SERPINA5	1.6×10^-20^	0.001
C6	9.6×10^-13^	0.488	SERPING1	1.7×10^-19^	1.6×10^-8^
C7	8.6×10^-19^	0.156	SERPINB3	1.3×10^-13^	0.175
C8A	0.008	0.416	SERPINA4	1.7×10^-14^	0.085
C8B	0.001	0.303	SERPINF2	1.2×10^-11^	0.03
C8G	0.025	0.069	SERPINA6	9.5×10^-18^	0.015
C9	1.0×10^-5^	0.833	SERPINA7	0.86	0.933
CD55	5.1×10^-20^	0.006	SERPIND1	4.0×10^-5^	0.067
CD59	8.0×10^-7^	0.008	SERPINB6	5.2×10^-4^	0.322
CD93	6.3×10^-17^	0.159	SERPINB5	3.0×10^-6^	0.159

IgAN, immunoglobulin A nephropathy; HC, health controls; MN, membranous nephropathy.

**Figure 3:**
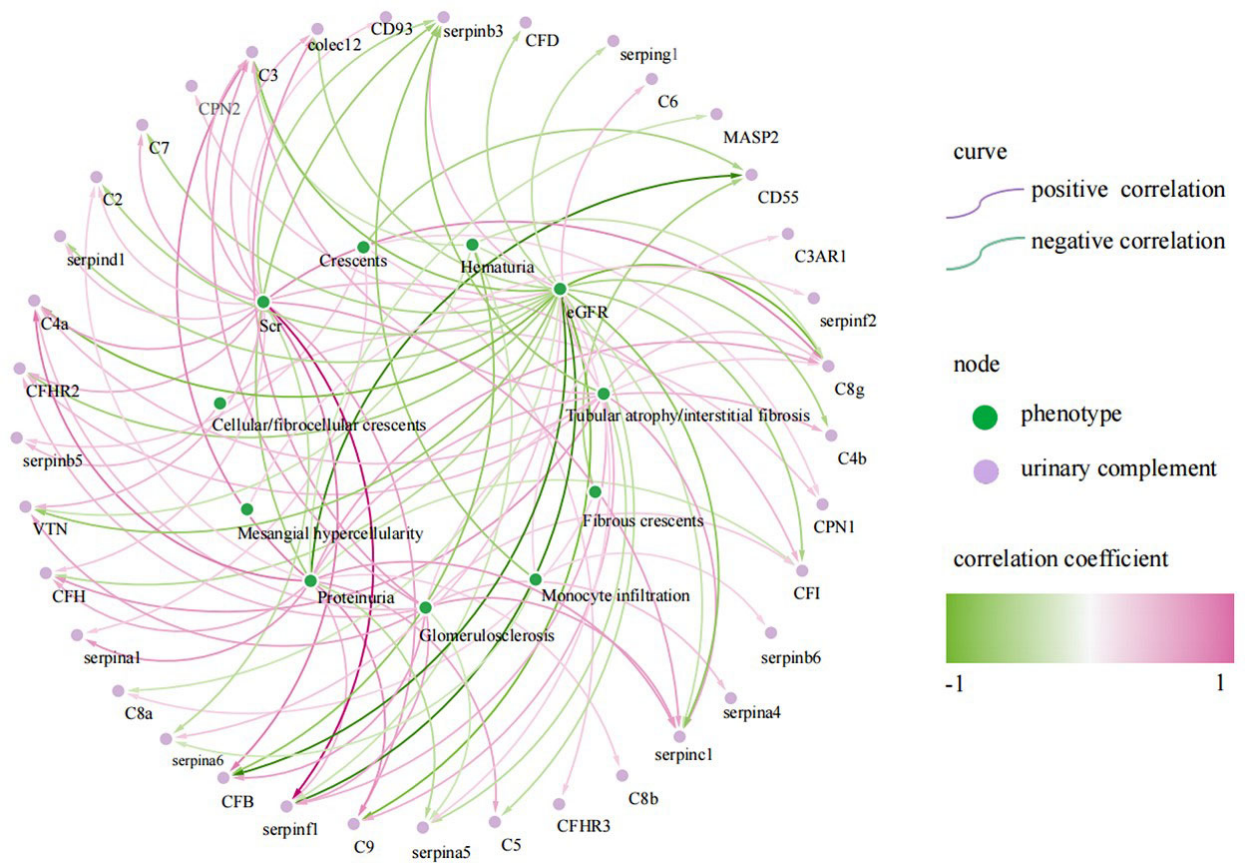
Correlation of urinary complement-related proteins with laboratory and pathological indices in patients with IgA nephropathy(IgAN) (only curves with p-values <0.05 are shown). The purple curves represent a positive correlation and the green curves represent a negative correlation, the darker the color, the stronger the correlation. The green nodes represent phenotypes of IgAN, the purple nodes represent urinary complement-related proteins.

We further analyzed the relationship between urine complement proteins and clinical or pathological parameters. The 24 h proteinuria was significantly positively correlated with the abundances of 12 urinary complements, including CFH (r = 0.400, *P* < 0.05) and C2 (r = 0.286, *P* < 0.05), and negatively correlated with the abundances of 5 urinary complements such as CD55 (r = -0.623, *P* < 0.05). EGFR was significantly negatively correlated with the abundances of 19 urinary complements including CFB (r = -0.654, *P* < 0.05) and collectin12 (colec12) (r = -0.380, *P*<0.05), and positively correlated with the abundances of 4 urinary complements. Serum creatinine (Scr) was significantly positively correlated with the abundances of 15 urinary complements, such as C4a (r = 0.443, *P* < 0.05), colec12 (r = 0.376, *P* < 0.05) and CFB (r = 0.572, *P* < 0.05), and negatively correlated with the abundances of 2 urinary complements. Hematuria (expressed by RBC counts per high-power field) was significantly negatively correlated with the abundances of 7 urinary complements, such as CFB (r = -0.480, *P* < 0.05) (**Figure 3**).

Cellular/fibrocellular crescents were positively correlated with urinary complement C6 (r = 0.342, *P* < 0.05), CPN1 (r = 0.317, *P* < 0.05) and SERPINB5 (r = 0.362, *P* < 0.05), and negatively correlated with urinary complement CD55 (r = -0.41, *P* < 0.05). Glomerulosclerosis was positively correlated with the abundances of 11 urinary complements, including C4a (r = 0.347, *P* < 0.05), colec12 (r = 0.463, *P* < 0.05), CFB (r = 0.446, *P* < 0.05) and CFHR2 (r = 0.371, *P* < 0.05), and negatively correlated with the abundances of urinary complement SERPINA5 (r = -0.294, *P* < 0.05). Tubular atrophy/interstitial fibrosis (TIF) was positively correlated with the abundances of 8 urinary complements, such as C4a (r = 0.356, *P* < 0.05), CFH (r = 0.473, *P* < 0.05) and CFHR2 (r = 0.355, *P* < 0.05), and negatively correlated with the abundances of 2 urinary complements (**Figure 3**).

We compared the urinary complement components between patients with proteinuria < or ≥ 1 g/24h. Multiple components are significantly higher in those with proteinuria ≥ 1 g/24h, including C2 (representing the classical pathway), CFH (representing the alternative pathway), C3 (representing the common pathway), C8g and C9 (terminal complement activation product) (**Supplementary Table 1**). We further compared the clinical parameters between IgAN patients with higher and lower urinary MASP2 levels (divided by MASP2 intensity = 0.00018), and found that the patients with lower urinary MASP2 had significantly higher proteinuria (**Supplementary Table 2**).

### Validation of the correlation between urine complement proteins and clinical or pathological parameters in IgAN patients

We further validated 7 complement proteins in the validation cohorts, including C4a, CD55, CFH, CFHR2, colec12, C6, and CFB, by ELISA (**Table 3**). Urine C4a/Cr (r = 0.323, *P* < 0.05), CFH/Cr (r = 0.330, *P* < 0.05), CFHR2/Cr (r = 0.375, *P* < 0.05), colec12/Cr (r = 0.589, *P* < 0.05), CFB/Cr (r = 0.362, *P* < 0.05) were positively correlated with 24 h proteinuria; CFH/Cr (r = -0.367, *P* < 0.05), CFHR2/Cr (r = -0.337, *P* < 0.05), and CFB/Cr (r = -0.300, *P* < 0.05) were negatively correlated with eGFR; CFB/Cr (r = 0.307, *P* < 0.05) was positively correlated with serum creatinine. However, there was a positive correlation between urinary complement-related protein CD55 (r = 0.368, *P* < 0.05) and 24 h proteinuria, which was different from the trend of urinary proteomics.

**Table 3:** Correlation analysis of seven urinary complement-related proteins with laboratory and pathological parameters in patients with IgAN.

	Urine complements (ng/mg)							
	CD55/Cr	CFH/Cr		CFHR2/Cr	colec12/Cr	C4a/Cr	CFB/Cr	C6/Cr
Serum uric acid (mmol/l)	r=-0.041	r=0.207		r=0.246	r=-0.095	r=-0.075	r=0.158	r=-0.012
Serum C3 (g/L)	r=-0.244	r=-0.039		r=0.004	r=-0.1115	r=-0.097	r=-0.133	r=-0.156
Serum creatinine (µmol/L)	r=0.228	r=0.235		r=0.289	r=0.039	r=0.1148	r=0.307*	r=0.164
eGFR (mL/min/1.73 m^2^)	r=-0.112	r=-0.367*		r=-0.337*	r=-0.242	r=-0.17	r=-0.300*	r=-0.264
Proteinuria (g/24h)	r=0.368*	r=0.330*		r=0.375*	r=0.589*	r=0.323*	r=0.362*	r=0.034
Hematuria	r=0.170	r=0.081		r=0.132	r=-0.055	r=0.054	r=0.255	r=-0.036
Fibrous crescents (%)	r=0.156	r=0.247		r=0.049	r=0.366*	r=0.202	r=0.233	r=0.204
Cellular/fibrocellular crescents (%)	r=0.097	r=-0.144		r=-0.039	r=0.104	r=0.130	r=-0.106	r=0.096
TIF (%)	r=0.123	r=0.450*		r=0.340*	r=0.067	r=0.102	r=0.394*	r=0.145
Monocyte infiltration (%)	r=0.186	r=0.195		r=0.182	r=0.266	r=0.272	r=0.350*	r=0.039

IgAN, immunoglobulin A nephropathy; eGFR, estimated glomerular filtration rate; TIF, Tubular atrophy/interstitial fibrosis. * p < 0.05.

CFH/Cr (r = 0.450, *P* < 0.05), CFHR2/Cr (r = 0.340, *P* < 0.05), and CFB/Cr (r = 0.394, *P* < 0.05) were positively correlated with tubular atrophy/interstitial fibrosis, CFB/Cr (r = 0.35, *P* < 0.05) were positively correlated with monocyte infiltration. These results were consistent with urinary proteomic analysis. The significantly positive correlations between CFB and TIF or monocyte infiltration were not validated, they have similar positive trends (Data not shown). The colec12 was also demonstrated to be positively correlated with the fibrous crescent (r = 0.366, *P* < 0.05).

## Discussion

The complement system consists of nearly 60 proteins, which include proteins in three classical, lectin and alternative activation pathways (22), proteases interacting in a cascade-like fashion, multiple regulatory factors, and receptors (23). In this study, we used untargeted proteomic studies that first reported the urinary complement profile in IgAN. We detected 44 urinary complement proteins by LC–MS/MS and selected 7 complement proteins for validation by ELISA.

**Figure 4:**
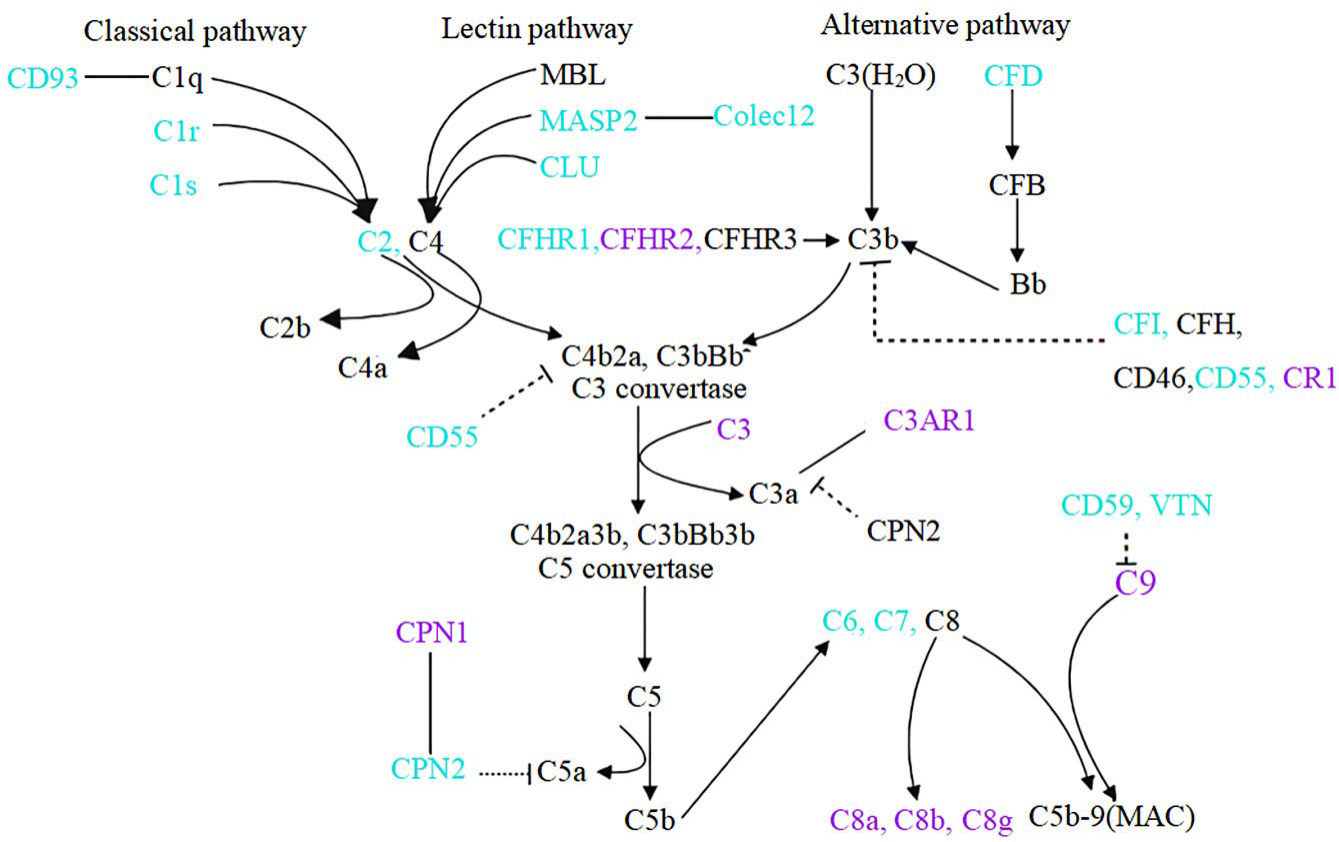
Components of complement system change in the urine of IgAN patients. Compared to healthy controls, blue font indicates down-regulation of urinary complement in IgAN patients, purple font indicates up-regulation, and black font indicates complement not detected or no statistically significant. Dotted lines indicate inhibitory relationships, solid arrows indicate facilitation, and solid lines without arrows indicate protein interactions.

The complement system serves as part of the innate immune system. In recent years, the role of the complement system in IgAN has attracted more attention (14, 16, 24, 25). It has been reported that glomerular MASP1/3 and MASP2 deposition was associated with crescent formation (26) and deposition of arteriolar C4d of the kidney was associated with progressive kidney disease in IgAN (14). Furthermore, the role of CFH and CFHRs in IgAN has attracted extensive attention from researchers (12, 16, 27, 28) since genome-wide association studies showed that deletion of the CFHR1-3 gene is highly protective in patients with IgAN (29, 30). Higher plasma CFHR5 levels were associated with severe histological features (15), proteinuria, hypertension, and reduced eGFR (12) in IgAN. The previous findings were mostly derived from serum or renal tissue, and only a few data from urine samples of IgAN patients. Changes in urine complement proteins could reflect complement overproduction, secretion, and/or deposition in the damaged kidney. In our study, we identified multiple up-regulated and down-regulated complement proteins in the urine of patients with IgAN (**Figure 4**).

Our results reveal that the urinary complement proteins of abnormal abundance are mainly enriched in the alternative and terminal pathways. A growing body of evidence implies that the activation of alternative pathways especially contributes to severe chronic lesions of kidney in IgAN patients (31). High abundance of urinary CFH and CFHR2, two members of the alternative pathway, is closely associated with glomerulosclerosis and TIF. It indicates that the activation of CFH and CFHR2 is a clue of the chronicity of IgAN. Our study agrees with previous studies (32, 33) and re-emphasizes the role of CFH and CFHRs in renal chronic damage in IgAN patients and their potential value as biomarkers of chronic injury. CFH is one of the most important circulating regulators of the alternative pathway and can accelerate the decay of the alternative pathway C3 convertase. Our results demonstrate that the CFH was positively correlated with 24h proteinuria and negatively correlated with eGFR. It is inconsistent with the results of CFH in serum. We speculate that the increase in proteinuria might be accompanied by an increase in urine CFH excretion (molecular weight of 155KDa), resulting in a decrease in circulatory CFH in IgAN patients. It has also been shown in previously published study (33). CFHR5 was an independent risk factor for IgAN progression (12, 15). CFHR genes are mainly transcribed in the liver and the proteins are distributed in plasma. CFHR proteins are emerging complement modulators, activators, and immune regulators (34). Each CFHR protein binds to C3b, iC3b, C3dg, and C3d. Several CFHR proteins interact with ligands such as glycosaminoglycans, monomeric CRP, pentraxin-3, malondialdehyde, and laminins of the kidney, and bind to the surfaces of apoptotic and necrotic cells (34). For the first time, we found a high abundance of CFHR2 in the urine of IgAN patients. It suggests that the liver might contribute to the overproduction of CFHR2, which is filtered from the glomeruli to the urine and could play an important role in the progression of IgAN. The evidence from previous and our studies indicates that complement activation is associated with the development of glomerular and tubulointerstitial injury.

The urinary CFB and C3 are correlated with hematuria, which suggests that CFB and C3 are associated with the activity of IgAN. In particular, C6 and CD55, which are associated with the cellular/fibrocellular crescents, indicate that IgAN is in pathological activity. Notably, our proteomic analysis showed a negative correlation between urinary CD55 and proteinuria, which is consistent with the study of Zhao et al. (35), suggesting a protective effect of CD55 on IgAN progression. Moreover, an interesting finding was that the difference of C1r and C1s were statistically significant between IgAN and healthy controls. Previous studies have shown that the lectin and alternative pathways were important in complement activation in patients with IgAN, but there was a lack of evidence for activation of the classical pathway. Our results from urine analysis imply the activation of C1r and C1s of the classic complement pathway in IgAN. It implied that the activation of classical pathway also participated in the pathogenesis of IgAN and need further study.

Collectin12, a member of the collectin family, is a pattern recognition molecule that initiates complement activation *via* the alternative pathway (36, 37). Collectin12 is coded by the collagen lectin gene *COLEC12* (38). Previous studies have shown that colec12 correlates with the severity of diabetic retinopathy (39), involving in myelin internalization (40) and promotes IL-23 expression by dendritic cells (41). Our study finds that urinary colec12 correlated with fibrous crescents and glomerulosclerosis in IgAN. It is the first report which demonstrates the renal chronic lesion of IgAN was related to urinary colec12.

Therapeutic complement regulation has attracted the attention of physicians. Several ongoing clinical trials targeting complements have included IgAN patients. The interventions targeting factor B in IgAN included small molecule LNP023 (NCT03373461), an antisense inhibitor of CFB mRNA IONIS-FB-LRx (NCT04014335). Moreover, a humanized monoclonal antibody to MASP-2, narsoplimab, is used to treat IgAN patients with persistent proteinuria (NCT03608033). There is an emerging need for complement-targeted therapy monitoring, and thus our study provides a highlight for future complement-targeted therapy and a potential basis for effective monitoring of complement interventions, particularly in IgAN.

An advantage of our study was the comprehensive quantification of urinary complement proteins using a targeted mass spectrometric method. It presents a profile of urinary complement protein in IgAN patients. The correlation between urinary complement proteins and clinical as well as pathological characteristics was analyzed. However, this study has several limitations. The origins of the complement proteins detected in urine were unclear. Moreover, we did not monitor the dynamic changes of the complements after the treatment of the IgAN by different agents.

In conclusion, multiple abnormally increased components of complement system were detected in the urine of IgAN patients, suggesting that the abnormal activation of complement system in IgAN patients is obvious. A high abundance of urinary complement proteins was significantly associated with clinical or pathological parameters. These components could be potential biomarkers for monitoring IgAN and provide clues for therapeutic choice targeting complement system of IgAN patients.

## Data availability statement

The mass spectrometry proteomics data have been deposited to the ProteomeXchange Consortium (http://proteomecentral.proteomexchange.org) via the iProX partner repository with the dataset identifier PDX038451.

## Ethics statement

The studies involving human participants were reviewed and approved by the Medical Ethics Committees at the Sichuan Provincial People’s Hospital. The patients/participants provided their written informed consent to participate in this study.

## Author contributions

DW designed and performed experiments, collected and analyzed data, interpreted experimental results and wrote the manuscript. CW collected the samples and clinical data. CW and YZ critiqued the experimental design. YZ performed liquid chromatography-tandem mass spectrometry (LC–MS/MS) analyses. DW performed ELISA experiments and conducted most of the experiments with help from SC, YL, and LW. GL designed the project, led the research team, analyzed data, and critiqued the manuscript. All authors have reviewed the manuscript. All authors contributed to the article and approved the submitted version.
