# Supplementary material for: Urinary complement profile in IgA nephropathy and its correlation with the clinical and pathological characteristics
Source: Front Immunol. 2023 Mar 20;14:1117995. doi: 10.3389/fimmu.2023.1117995 (PMC10068869; doi:10.3389/fimmu.2023.1117995)
Supplement: Supplementary file 1 [file DataSheet_1.pdf]

Supplemental Figures

Supplemental **Figure S1.**

Figure S1. Gene Ontology bubble plots of urinary differentially expressed proteins between IgA nephropathy (IgAN) and healthy controls (HC). Gene Ontology (GO) bubble plots of urinary differentially expressed proteins in biological process (BP), cellular component (CC), and molecular function (MF) between IgAN and HC, respectively. (A) Urinary differentially expressed proteins up-regulated in BP; (B) Urinary differentially expressed proteins up-regulated in CC; (C) Urinary differentially expressed proteins up-regulated in MF; (D) Urinary differentially expressed proteins down-regulated in BP; (E) Urinary differentially expressed proteins down-regulated in CC; (F) Urinary differentially expressed proteins down-regulated in MF.

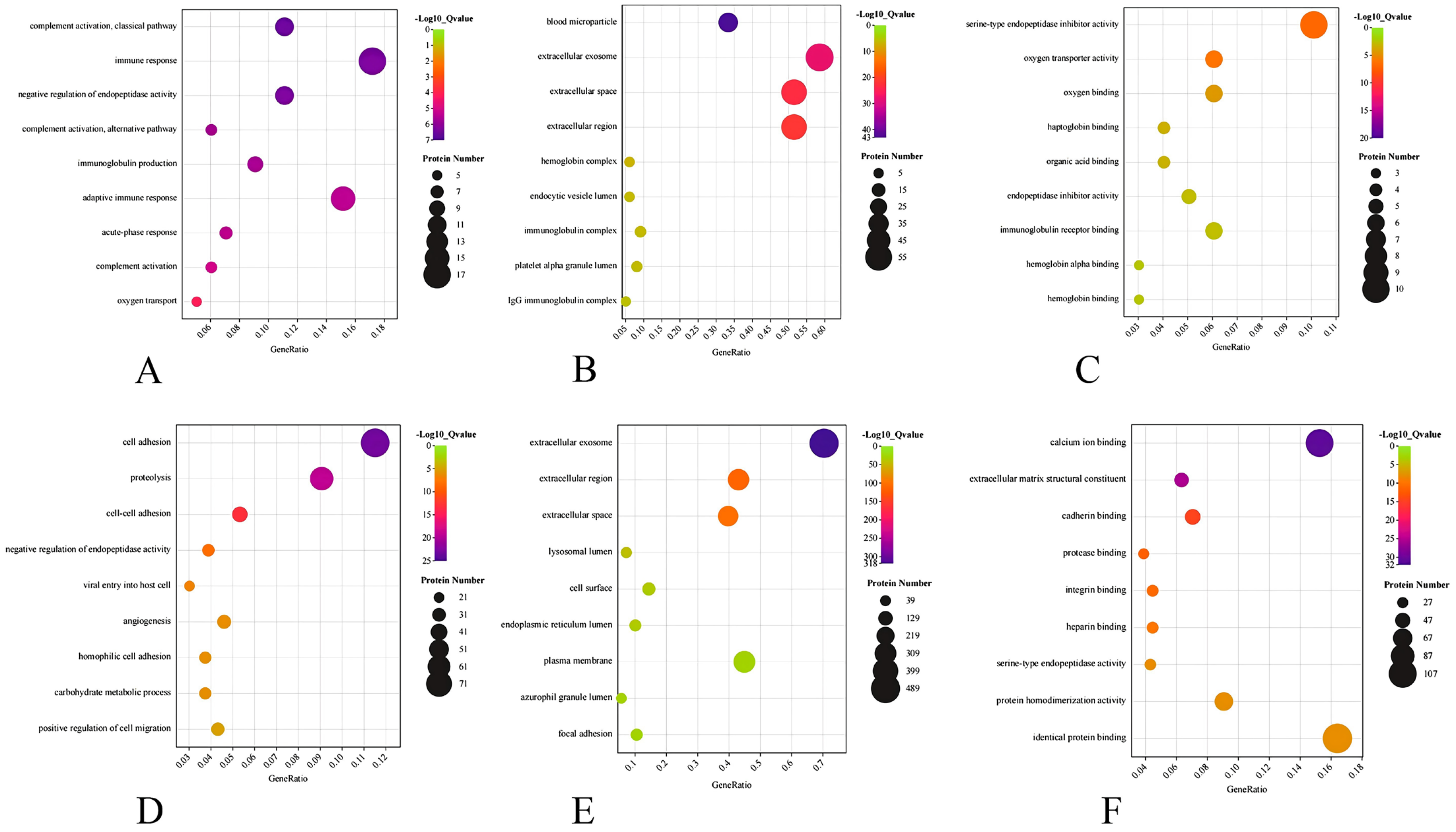
complement activation, classical pathway
immune response
negative regulation of endopeptidase activity
complement activation, alternative pathway
immunoglobulin production
adaptive immune response
acute-phase response
complement activation
oxygen transport
GeneRatio
-Log10_Qvalue
Protein Number
A
blood microparticle
extracellular exosome
extracellular space
extracellular region
hemoglobin complex
endocytic vesicle lumen
immunoglobulin complex
platelet alpha granule lumen
IgG immunoglobulin complex
GeneRatio
-Log10_Qvalue
Protein Number
B
serine-type endopeptidase inhibitor activity
oxygen transporter activity
oxygen binding
haptoglobin binding
organic acid binding
endopeptidase inhibitor activity
immunoglobulin receptor binding
hemoglobin alpha binding
hemoglobin binding
GeneRatio
-Log10_Qvalue
Protein Number
C
cell adhesion
proteolysis
cell-cell adhesion
negative regulation of endopeptidase activity
viral entry into host cell
angiogenesis
homophilic cell adhesion
carbohydrate metabolic process
positive regulation of cell migration
GeneRatio
-Log10_Qvalue
Protein Number
D
extracellular exosome
extracellular region
extracellular space
lysosomal lumen
cell surface
endoplasmic reticulum lumen
plasma membrane
azurophil granule lumen
focal adhesion
GeneRatio
-Log10_Qvalue
Protein Number
E
calcium ion binding
extracellular matrix structural constituent
cadherin binding
protease binding
integrin binding
heparin binding
serine-type endopeptidase activity
protein homodimerization activity
identical protein binding
GeneRatio
-Log10_Qvalue
Protein Number
F
