# Supplementary material for: Urinary complement profile in IgA nephropathy and its correlation with the clinical and pathological characteristics
Source: Front Immunol. 2023 Mar 20;14:1117995. doi: 10.3389/fimmu.2023.1117995 (PMC10068869; doi:10.3389/fimmu.2023.1117995)
Supplement: Supplementary file 2 [file Table_1.docx]

**Supplementary Table1.** Subgroup analysis of urinary complement levels in IgAN patients with 24h proteinuria <1g or ≥1g.

| Characteristics | Proteinuria < 1g/24h | Proteinuria ≥ 1g/24h | HC | *P* Value^#^ | *P* Value^&^ | | | *P* Value* |
| --- | --- | --- | --- | --- | --- | --- | --- | --- |
| Number n(%) | 9(18%) ^c^ | 41(82%) ^c^ |  |  | |  |  | |
| Gender (male, %) | 3(33%) ^c^ | 22(54%) ^c^ |  |  | |  | NS | |
| Age (yr) | 26(20, 44) ^c^ | 37(26, 47) ^c^ |  |  | |  | NS | |
| Percentage of crescents of all patients (%) | 2(22%) ^c^ | 25(61%) ^c^ |  |  | |  | ＜0.05 | |
| Percentage of cellular/fibrocellular crescents of all patients (%) | 2(22%) ^c^ | 19(46%) ^c^ |  |  | |  | NS | |
| Percentage of fibrous crescents of all patients (%) | 0(0%) ^c^ | 15(37%) ^c^ |  |  | |  | ＜0.05 | |
| Mesangial hypercellularity n (%) | 5(56%) ^c^ | 31(76%) ^c^ |  |  | |  | NS | |
| Percentage of glomerulosclerosis of all patients (%) | 3(33%) ^c^ | 18(44%) ^c^ |  |  | |  | NS | |
| Percentage of monocyte infiltration of all patients (%) | 5(56%) ^c^ | 37(90%) ^c^ |  |  | |  | ＜0.05 | |
| Percentage of tubular atrophy/interstitial fibrosis of all patients (%) | 5(56%) ^c^ | 37(90%) ^c^ |  |  | |  | ＜0.05 | |
| C1R | 0(0, 6.05×10^-6^) | 0 | 1.81×10^-6^(0, 7.31×10^-6^) | NS | | ＜0.05 | NS | |
| C1S | 0 | 0 | 1.12×10^-5^(2.30×10^-7^, 2.53×10^-5^) | ＜0.05 | | ＜0.05 | NS | |
| C2 | 0 | 4.41×10^-6^(0, 2.27×10^-5^) | 3.83×10^-6^(0, 1.30×10^-5^) | ＜0.05 | | NS | ＜0.05 | |
| COLEC12 | 0(0, 2.60×10^-5^) | 0(0, 2.18×10^-5^) | 1.32×10^-4^(4.94×10^-5^, 2.16×10^-4^) | ＜0.05 | | ＜0.05 | NS | |
| CLU | 8.52×10^-4^(4.57×10^-4^, 1.12×10^-3^) | 7.77×10^-4^(4.88×10^-4^, 1.16×10^-3^) | 2.90×10^-3^(2.23×10^-3^, 5.20×10^-3^) | ＜0.05 | | ＜0.05 | NS | |
| MASP2 | 2.39×10^-4^(5.11×10^-5^, 3.20×10^-4^) | 1.65×10^-4^(6.60×10^-5^, 3.28×10^-4^) | 1.93×10^-3^(1.18×10^-3^, 3.65×10^-3^) | ＜0.05 | | ＜0.05 | NS | |
| C4A | 1.89×10^-4^(4.07×10^-5^, 5.79×10^-4^) | 1.46×10^-3^(3.70×10^-4^, 3.00×10^-3^) | 5.92×10^-4^(4.26×10^-4^, 8.15×10^-4^) | ＜0.05 | | ＜0.05 | ＜0.05 | |
| C4B | 3.44×10^-5^(0, 6.05×10^-5^) | 1.29×10^-4^(3.13×10^-5^, 2.23×10^-4^) | 9.85×10^-5^(4.63×10^-5^, 1.94×10^-4^) | ＜0.05 | | NS | ＜0.05 | |
| C3 | 3.64×10^-4^(1.46×10^-4^, 3.54×10^-3^) | 4.88×10^-3^(1.19×10^-3^, 7.42×10^-3^) | 2.09×10^-4^(9.98×10^-5^, 4.69×10^-4^) | NS | | ＜0.05 | ＜0.05 | |
| CFD | 0(0, 2.88×10^-6^) | 0(0, 9.74×10^-6^) | 9.25×10^-6^(1.41×10^-6^, 2.65×10^-4^) | ＜0.05 | | ＜0.05 | NS | |
| CFB | 8.72×10^-5^(1.98×10^-6^, 1.20×10^-4^) | 2.57×10^-4^(4.64×10^-5^, 6.57×10^-4^) | 1.28×10^-4^(9.11×10^-5^, 2.04×10^-4^) | ＜0.05 | | NS | NS | |
| CFI | 3.57×10^-5^(1.43×10^-5^, 5.39×10^-5^) | 5.32×10^-5^(2.30×10^-5^, 9.98×10^-5^) | 3.30×10^-4^(2.26×10^-4^, 6.01×10^-4^) | ＜0.05 | | ＜0.05 | NS | |
| CFH | 0(0, 3.28×10^-6^) | 7.69×10^-6^(2.43×10^-7^, 4.54×10^-5^) | 1.28×10^-5^(6.39×10^-6^, 2.81×10^-5^) | ＜0.05 | | NS | ＜0.05 | |
| CFHR1 | 0(0, 7.87×10^-5^) | 0 | 5.16×10^-5^(2.32×10^-5^, 9.67×10^-5^) | ＜0.05 | | ＜0.05 | ＜0.05 | |
| CFHR2 | 0 | 0 | 0(0, 5.38×10^-6^) | ＜0.05 | | ＜0.05 | NS | |
| CFHR3 | 0 | 0 | 0 | NS | | NS | NS | |
| C5 | 0 | 0 | 0 | NS | | NS | NS | |
| C6 | 0 | 0 | 3.64×10^-6^(1.49×10^-6^, 1.07×10^-5^) | ＜0.05 | | ＜0.05 | NS | |
| C7 | 4.08×10^-5^(2.12×10^-5^, 7.56×10^-5^) | 1.37×10^-5^(0, 6.58×10^-5^) | 2.99×10^-4^(2.29×10^-4^, 3.95×10^-4^) | ＜0.05 | | ＜0.05 | NS | |
| C8A | 0 | 0(0, 8.61×10^-6^) | 0 | NS | | ＜0.05 | NS | |
| C8B | 0 | 0 | 0 | NS | | ＜0.05 | NS | |
| C8G | 0 | 0(0, 1.32×10^-5^) | 0(0, 1.63×10^-6^) | NS | | ＜0.05 | ＜0.05 | |
| C9 | 4.41×10^-5^(0, 1.18×10^-4^) | 1.76×10^-4^(4.33×10^-5^, 6.00×10^-4^) | 2.73×10^-5^(6.91×10^-6^, 6.11×10^-5^) | NS | | ＜0.05 | ＜0.05 | |
| C4BPA | 0 | 0 | 0 | NS | | ＜0.05 | NS | |
| C3AR1 | 0 | 0 | 0 | NS | | ＜0.05 | NS | |
| CPN1 | 0(0, 6.30×10^-6^) | 0(0, 7.34×10^-6^) | 0 | ＜0.05 | | ＜0.05 | NS | |
| CPN2 | 7.44×10^-5^(4.43×10^-5^, 1.22×10^-4^) | 5.29×10^-5^(1.96×10^-5^, 1.02×10^-4^) | 3.11×10^-4^(1.54×10^-4^, 4.25×10^-4^) | ＜0.05 | | ＜0.05 | NS | |
| CD55 | 8.83×10^-4^(2.62×10^-4^, 1.16×10^-3^) | 7.57×10^-5^(2.61×10^-5^, 2.45×10^-4^) | 3.04×10^-3^(2.15×10^-3^, 4.11×10^-3^) | ＜0.05 | | ＜0.05 | ＜0.05 | |
| CD59 | 1.14×10^-3^(1.01×10^-3^, 1.81×10^-3^) | 7.55×10^-4^(2.92×10^-4^, 1.22×10^-3^) | 1.38×10^-3^(1.05×10^-3^, 1.99×10^-3^) | NS | | ＜0.05 | ＜0.05 | |
| CD93 | 0 | 0 | 8.76×10^-6^(4.82×10^-6^,1.31×10^-5^) | ＜0.05 | | ＜0.05 | NS | |
| VTN | 2.21×10^-4^(1.26×10^-4^, 3.60×10^-4^) | 2.22×10^-4^(5.41×10^-5^, 5.61×10^-4^) | 7.00×10^-4^(5.55×10^-4^, 8.96×10^-4^) | ＜0.05 | | ＜0.05 | NS | |
| SERPINA1 | 4.57×10^-2^(2.46×10^-2^, 6.36×10^-2^) | 6.74×10^-2^(5.37×10^-2^, 8.24×10^-2^) | 2.74×10^-3^(2.13×10^-3^, 3.82×10^-3^) | ＜0.05 | | ＜0.05 | ＜0.05 | |
| SERPINA3 | 8.20×10^-3^(4.42×10^-3^, 1.05×10^-2^) | 6.12×10^-3^(4.99×10^-3^, 8.30×10^-3^) | 1.53×10^-3^(8.92×10^-4^, 2.83×10^-3^) | ＜0.05 | | ＜0.05 | NS | |
| SERPINA4 | 0(0, 4.97×10^-6^) | 0(0, 5.57×10^-6^) | 4.42×10^-5^(2.19×10^-5^, 7.21×10^-5^) | ＜0.05 | | ＜0.05 | NS | |
| SERPINA5 | 5.01×10^-5^(0, 8.98×10^-5^) | 1.11×10^-5^(0, 3.48×10^-5^) | 1.23×10^-3^(9.05×10^-4^, 2.01×10^-3^) | ＜0.05 | | ＜0.05 | NS | |
| SERPINA6 | 3.75×10^-3^(2.10×10^-3^, 5.31×10^-3^) | 3.13×10^-3^(1.88×10^-3^, 4.77×10^-3^) | 5.83×10^-4^(3.57×10^-4^, 8.88×10^-4^) | ＜0.05 | | ＜0.05 | NS | |
| SERPINB3 | 2.23×10^-5^(2.65×10^-6^, 1.95×10^-4^) | 0 | 2.31×10^-4^(1.65×10^-5^, 5.05×10^-4^) | ＜0.05 | | ＜0.05 | ＜0.05 | |
| SERPINB5 | 0 | 0 | 0(0, 1.92×10^-5^) | ＜0.05 | | ＜0.05 | NS | |
| SERPINB6 | 0(0, 6.05×10^-6^) | 0 | 0(0, 1.18×10^-6^) | NS | | ＜0.05 | NS | |
| SERPINC1 | 0 | 1.99×10^-3^(8.29×10^-4^, 3.69×10^-3^) | 2.47×10^-4^(1.53×10^-4^, 3.94×10^-4^) | ＜0.05 | | ＜0.05 | ＜0.05 | |
| SERPIND1 | 0 | 0(0, 3.01×10^-6^) | 3.03×10^-6^(1.54×10^-7^, 6.27×10^-6^) | ＜0.05 | | ＜0.05 | NS | |
| SERPINF1 | 0(0, 2.60×10^-5^) | 7.27×10^-5^(2.85×10^-5^, 2.31×10^-4^) | 7.77×10^-5^(4.17×10^-5^, 1.15×10^-4^) | ＜0.05 | | NS | NS | |
| SERPINF2 | 8.52×10^-4^(4.57×10^-4^, 1.12×10^-3^) | 5.58×10^-5^(1.55×10^-5^, 8.16×10^-5^) | 1.57×10^-4^(1.08×10^-4^, 2.18×10^-4^) | ＜0.05 | | ＜0.05 | NS | |
| SERPING1 | 2.39×10^-4^(5.11×10^-5^, 3.20×10^-4^) | 5.06×10^-4^(2.88×10^-4^, 6.18×10^-4^) | 3.12×10^-3^(2.16×10^-3^, 4.46×10^-3^) | ＜0.05 | | ＜0.05 | ＜0.05 | |

Accreviations: HC: healthy control. NS: not statistically. *c:* Data are expressed as frequency and ratio. #: Proteinuria < 1g/24h vs HC. & : Proteinuria ≥ 1g/24h vs HC. * : Proteinuria < 1g/24h vs Proteinuria ≥ 1g/24h).

**Supplementary Table2.**  Subgroup analysis of MASP2 activation (lectin pathway）in IgAN patients

| Characteristics | Intensity of MASP2  ＜0.00018^d^ | | Intensity of MASP2  ≥0.00018 ^d^ | | *P* Value |  |
| --- | --- | --- | --- | --- | --- | --- |
|  |  |  |  |  |  |  |
| Gender (male, %) | | 13(52%) ^c^ | 12(48%) ^c^ | NS | |  |
| Age (yr) | | 36(25, 47) ^b^ | 33(24, 47) ^b^ | NS | |  |
| Hypertension (mmHg) | | 7(28%) ^c^ | 8(32%) ^c^ | NS | |  |
| Serum creatinine (μmol/L) | | 87.5(62.5, 128.8) ^b^ | 90.6(62.35, 127.95) ^b^ | NS | |  |
| eGFR (mL/min/1.73m^2^) | | 78.97(56.68, 114.55) ^b^ | 90.26(57.77,113.90) ^b^ | NS | |  |
| Proteinuria (g/24h) | | 3.08(1.563, 4.3) ^b^ | 1.56(1.00, 2.76) ^b^ | ＜0.05 | |  |
| Hematuria | | 29.05(5.005, 85.155) ^b^ | 14.49(3.85, 76.51) ^b^ | NS | |  |
| Crescents (%) | | 14(56%) ^c^ | 13(52%) ^c^ | NS | |  |
| Cellular/fibrocellular  crescents n (%) | | 10(40%) ^c^ | 11(44%) ^c^ | NS | |  |
| Fibrous crescents n (%) | | 8(32%) ^c^ | 7(28%) ^c^ | NS | |  |
| Mesangial hypercellularity n (%) | | 19(76%) ^c^ | 17(68%) ^c^ | NS | |  |
| Glomerulosclerosis n (%) | | 12(48%) ^c^ | 9(36%) ^c^ | NS | |  |
| Monocyte infiltration n (%) | | 24(96%) ^c^ | 18(72%) ^c^ | NS | |  |
| Tubular atrophy/interstitial fibrosis n (%) | | 20(80%) ^c^ | 22(88%) ^c^ | NS | |  |

Accreviations: MASP2: mannose associated serine protease 2; eGFR: estimated glomerular filtration rate; Hematuria: expressed by RBC counts per high-power field. *b:* Data are expressed as medians and interquartile ranges (IQRs). *c:* Data are expressed as frequency and ratio. *d:* the medians of MASP2 intensity. NS: not statistically.
